# Access to diagnostic testing for invasive fungal diseases and other opportunistic infections in Mexican health care centers caring for patients living with HIV

**DOI:** 10.1186/s12913-025-12405-5

**Published:** 2025-02-19

**Authors:** Dora E. Corzo-Leon, Nancy Martinez-Rivera, Alexandra Martin-Onraet, Alicia Piñeirua-Menendez

**Affiliations:** 1 MRC Centre for Medical Mycology, University of Exeter, Geoffrey Pope Building, Stocker Road, Exeter, EX4 4QD UK; 2 Infectious Diseases Department, Instituto Nacional de Cancerologia, Mexico City, Mexico; 3 CISIDAT, Health Research Consortium, Cuernavaca, Mexico

**Keywords:** HIV-related opportunistic infections, Rapid diagnostic tests, Latin America, Invasive mycoses

## Abstract

**Background:**

The burden of opportunistic infections (OIs) remains high among people living with HIV (PLWH) in Mexico, despite improvements in mortality worldwide.

**Objective:**

Reporting the current access to diagnostics of OIs in Mexican Health Care Centers offering health-care services to PLWH.

**Methods:**

An online questionnaire was sent to public health care facilities providing HIV care in Mexico. We evaluated capacities to 1) identify individuals with advanced-HIV, and 2) local and/or on-site access to: point-of-care assays, imaging studies, histological analysis, and microbiology tests useful to diagnose a wide variety of OIs.

**Results:**

In 2022, 46 centers answered the questionnaire, from 23/32 (71.8%) states of the country; 29 (63%) were primary care facilities, 5 (11%) general hospitals and 12 (26%) tertiary care hospitals, providing health services to 67,000 PLWH. These centers received 1,135 new patients/month, 48% with advance disease. Less than 50% could determine CD4 + T cell count (39%, *N* = 18), toxoplasma serology (41%, *N* = 19) and HIV viral load (41%, *N* = 19). Twenty-five centers could diagnose cryptococcosis and tuberculosis (54%). Meanwhile, 11 centers (24%) had access to aspergillosis or *Histoplasma* tests. Seven centers (11%) had access to coccidioidomycosis tests, five centers to any *Pneumocystis* diagnosis. In primary care centers, *Mycobacterium tuberculosis* complex GeneXPert was accessible in 41%, cryptococcal antigen by latex agglutination was available in one facility (3%), Indian ink in 9 centers (31%). No primary health center had access to lateral flow test for *Cryptococcus* or *Histoplasma* antigens.

**Conclusions:**

In Mexico, most public HIV-dedicated health care centers lack on-site capacity to diagnose opportunistic infections, specifically fungal infections. Rapid tests and point-of-care tests are frequently unavailable, which is more pronounced in primary care centres. Considering that IFDs still contribute significantly to mortality among PLWH, better access to diagnostic tools in all levels of HIV-care is urgent.

**Supplementary Information:**

The online version contains supplementary material available at 10.1186/s12913-025-12405-5.

## Background

Worldwide, there have been significant improvements in mortality and life expectancy among people living with HIV (PLWH) over the past few decades [1–3]. However, advanced disease still represents approximately 43% of new HIV diagnosis in Mexico [4]. The burden of opportunistic infections (OIs) among PLWH experiencing advanced disease remains high and is driven mainly by invasive fungal diseases (IFDs) and mycobacterial diseases [5]. IFD contribute to almost 50% of mortality causes in PLWH [5–8]. The diagnosis of IFDs is challenging, since it requires access to infrastructure and/or tests that are not widely available [9]. These diseases are underdiagnosed worldwide, predominantly in low- and middle-income countries (LMICs) [10]. The World Health Organization (WHO) recently issued the first fungal priority pathogen list, where *Cryptococcus neoformans* is ranked as critical priority pathogen along with *Candida auris*, *Aspergillus fumigatus* and *Candida albicans*. Meanwhile, *Histoplasma* spp. was ranked as a high priority pathogen and *Coccidioides* spp. as medium priority [11]. The areas of action proposed by this priority list require focus on improving fungal diagnostic capacity. The report encourages countries to invest in research and adopt public policies to strengthen their laboratory and surveillance capacities. These policy changes and research investments should be based on updated local data on each country's situation. Local data on the laboratory capacities, aimed to improve the identification of OIs, including IFDs is lacking in Mexico. In this study, we evaluated the current access to OIs diagnostic methods in Mexican Health Care Centers offering HIV care. This study serves as a first step to identify health care needs that will lead to improving local diagnostic capacities.

## Methods

### Local context

Mexico has a complicated and fragmented health system, with at least three components operating: 1) employment-based social security managed by national institutions (Instituto Mexicano de Seguro Social (IMSS), Instituto de Seguridad y Servicios Sociales de Trabajadores del Estado (ISSSTE)) 2) public non-employment-based services and 3) private sector (private services and insurances). Such systems have no interconnection or communication among them [12]. Patients with no formal labor or non-employment-based insurance are covered by the local state or federal Health Ministry. This specific coverage will be referred to as public sector. By the end of 2022, Mexican Health Ministry reported approximately 350,628 persons live with HIV in Mexico [4]. The public sector in Mexico provides health care to approximately 60% of these PLWH, an estimate of 210,376 persons. The public sector provides HIV care mainly through CAPASITS, which are primary healthcare units specialized on HIV, viral hepatitis and other sexual transmitted diseases.

### Procedures

An online questionnaire was designed for this study based on previous surveys conducted in Asia, Europe and Africa [9, 13, 14]. From January to December 2022, this questionnaire collected information about laboratory capacities among public health care centers providing PLWH care in the public sector, including primary and secondary health-care facilities as well as tertiary care hospitals in Mexico (Suppl 1: questionnaire). The questionnaire was sent via email to either the director and/or the local infectious diseases/medical doctor of all primary care facilities of each state, and to at least one general hospital or tertiary care hospital of each of the 32 Mexican states. The survey collected information about the number of PLWH receiving medical care at each of the consulted health facilities every month and the proportion of patients with advanced disease (< 200 CD4 + T cells/mm3 at diagnosis) in the previous years (2020–21). Laboratory capacities evaluated by the same questionnaire were: 1) identification of individuals at higher risk of opportunistic infections (OIs) through access to CD4 + T cell count measurements, 2) local and/or on-site access to imaging studies (CT scan, X-ray), histological analysis and microbiology tests (microscopy, cultures, serology, and biomarkers for a wide variety of OIs such as tuberculosis, fungal infections, syphilis, hepatitis and, toxoplasmosis).

### Analysis

To define the capacity of each facility to diagnose histoplasmosis, cryptococcosis, pneumocystosis, coccidioidomycosis, aspergillosis, and tuberculosis, we classified all diagnostic tests according to their potential to diagnose each of these OIs. Access to viral hepatitis and toxoplasmosis serologies by the participant facilities was also considered in our definition. Laboratory capacity was analyzed by type of centers (primary, secondary and tertiary care) and was mapped for each state. Maps were created with the information obtained from each center to describe the local capacity to diagnose a specific OI in each state. A state was considered having diagnostic capacities if at least one participant center in that state reported having local diagnostic tools for each OI. We also mapped states according to their proportion of individuals with advanced disease diagnosis (CD4 + T cell count ≤ 200 cell/mm3). These states were categorized as those with ≤ or ≥ 50% of PLWH with advanced disease. Univariate analysis was also performed by comparing the diagnostic capacity among states considering the proportion of advanced HIV diagnosis. Analysis was performed with statistical software package STATA 14·0.

### Ethics

This research was considered minimal risk according to local regulations set by the Reglamento de la Ley General de Salud en Materia de Investigación para la Salud so ethical approval and informed consent were waived.

## Results

**Fig. 1:**
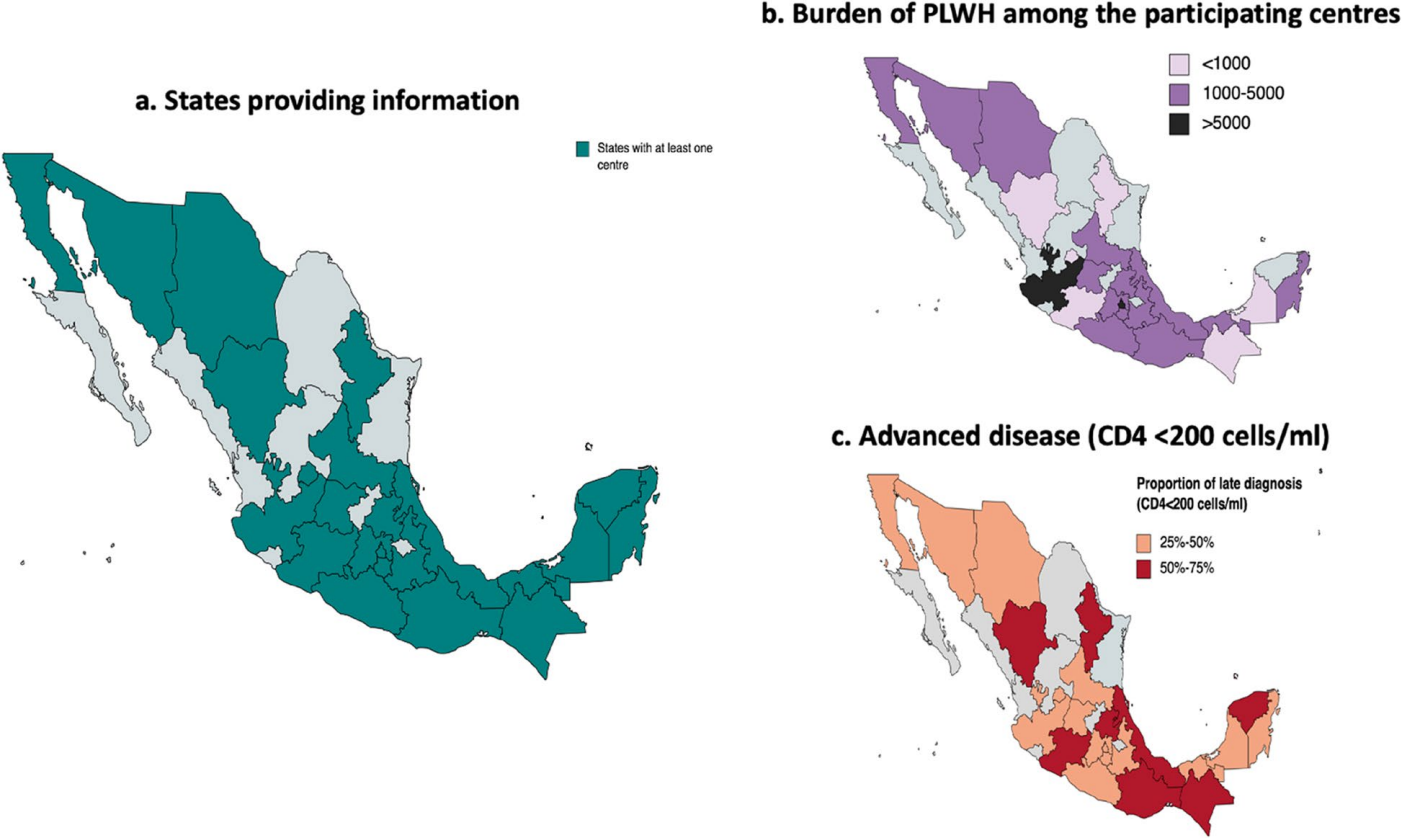
Distribution and characteristics of centers providing information, by state. **a** States providing information (at least one center): Number of centers per state and % of total centers. Aguascalientes: 2 (4%), Baja California: 2 (4%), Campeche: 1 (2%), CDMX: 6 (11%), Chiapas: 3 (6%), Chihuahua: 1 (2%), Durango: 1(2%), Estado de México: 2 (4%), Guanajuato: 9 (16%), Guerrero: 1 (2%), Hidalgo: 1 (2%), Jalisco: 3 (6%), Michoacán: 1 (2%), Morelos: 2 (4%), Nuevo León: 1 (2%), Oaxaca: 2 (4%), Puebla: 1 (2%), Quintana Roo: 5 (9%), San Luis Potosí: 1 (2%), Sonora 5 (9%), Tabasco: 1 (2%), Veracruz: 2 (4%), Yucatán: 1 (2), **b** attention burden (total number of registered patients) and **c** proportion of patients diagnosed in advanced disease (CD4 < 200 cells/ml) per state. Maps created with mapchart.net

The survey was sent via email to 182 health care providers working in primary, secondary and/or tertiary care centres where HIV care is provided. We received 104 answers (57·1%). After screening for repeated answers corresponding to the same center, 46 answers were selected corresponding to information of 54 centers. These centers are distributed among 23 of the 32 states in the country, accounting for 72% of the total Mexican territory (Fig. 1a). These states include the HIV-dedicated institutions with the highest number of patients in the country, located in Mexico City, Guadalajara and Monterrey. For most states, we received three or less responses: 11 of the 23 states sent one answer per state, six states with two answers per state and two states with three answers per state. Four states had five or more answers. Altogether, these centers provided health services to 67,003 PLWH, representing around one-third of all PLWH linked to care in the country [4].

Of the 46 answers, 29 (63%) were from primary care facilities (CAPASITS dedicated to HIV care), 5 (11%) were from general hospitals and 12 (26%) were tertiary care hospitals. The total number of PLWH reported receiving care monthly for all the centers was 15,101 patients. Most of the states reported providing care to between 1000 and 5000 patients. Only two states had cohorts larger than 5000 PLWH (Fig. 1b). Overall, these centers received 1,135 new patients every month (calculated as a monthly average of 2020 and 2021). Of those, 545 patients (48%) had advanced disease at diagnosis, hence considered at high risk of OI. Every center registered a median of 8 (1QR 4–14) new patients with advanced disease every month (Table 1). Eight states reported > 50% populations with advance diseases under their care (Fig. 1c). Among these eight states, only one state reported having access to rapid tests to diagnose the most important fungal infections.

**Table 1:** General characteristics and HIV profile of patients from participating health care centers

Characteristic	*N* = 46 (%)
Participants by type of health system, n (%)	
• Secretaria de Salud (Federal Health Ministry)	43 (94)
• Gobierno de la CDMX (Mexico City Health Ministry)	2 (4)
• University hospital	1 (2)
Healthcare level	
• First	29 (63)
• Second	5 (11)
• Third	12 (26)
Total PLWH registered in all centres (2020–2021)	67,003
Total PLWH attended every month by all centres	15,101
New registered PLWH for all centres (monthly average 2020–2021)	1,135
New PLWH (< 200 CD4 T-cells/mm^3^) for all centres (monthly average 2020–2021)	545
PLWH registered per centre, median (IQR)	773 (224 −1861)
PLWH attended per centre (monthly average 2020–2021), median (IQR)	200 (30–484)
New registered PLWH per centre (monthly average 2020–2021), median (IQR), *n* = 45	10 (5–23)
New PLWH (< 200 CD4 T-cells/mm^3^) per centre (monthly average 2020–2021), median (IQR)	7·5 (4–14)
*On site*/ local capacity of diagnosis of new PLHW cases and OI, *n* (%)	
• None	1 (2)
• Only new PLWH	14 (31)
• New PLWH and OI	31 (67)

*PLWH* People living with HIV, *CD4* Cluster of differentiation 4, *IQR* Interquartile range, *OI* Opportunistic infections

**Fig. 2:**
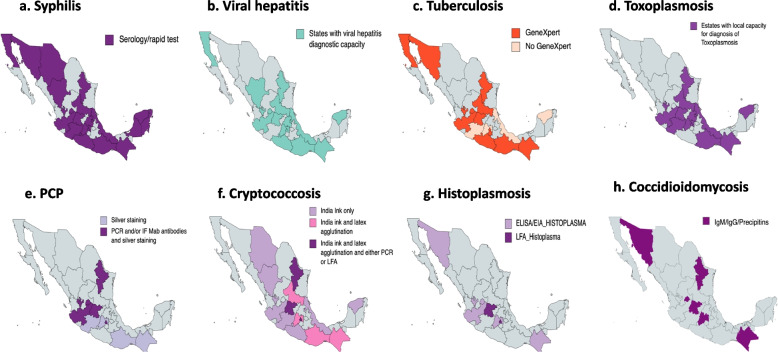
Map of local capacities to diagnose invasive fungal diseases and other opportunistic infections in Mexican Health Care Centers caring for patients living with HIV. Maps created with mapchart.net

Regarding all centers, < 50% had the local capacity to quantify CD4 + T cell count (39%, *N* = 18), toxoplasma serology (41%, *N* = 19) and HIV viral load (41%, *N* = 19) (Table 2). Sixty-five percent of all the centers (*N* = 30) could perform hepatitis serology and 78% had the rapid test for syphilis (Fig. 2a, b). Sixty-two percent (*N* = 28) of the centers reported having access to a microscope (Table 2). Of those, 82% were conventional microscopes. Eighty-two percent of the centers with microscopes used it, mostly for cytology (*N* = 23, 82%), Gram staining (*N* = 20, 71%), Ziehl Nielsen stain (*N* = 20, 71%) and KOH (*N* = 16, 57%) (Table 2). When a diagnostic service is not locally available (14 to 23 out of 46 centers), samples are sent to either an external centralized laboratory or a reference hospital. These external diagnostic services typically deliver results after 10 days (7–25 days) (Table 2).

**Table 2:** Laboratory resources available

Characteristic	*N* = 46 (%)
Centre has a clinical general laboratory *on site* (based on 46 responses)	25 (54)
If not clinical laboratory available *on site*, *n* = 23 (%)	
• Send samples to the state laboratory for analysis	11 (48)
• Send samples to a different reference hospital	12 (52)
Blood tests and assays carried out *on site*, *n* (%)	
• Complete blood count	37 (80)
• Glucose and kidney function	36 (78)
• Liver function testing	32 (69)
• CD4 T cell count	18 (39)
• HIV viral load	19 (41)
• Toxoplasma serology (IgM/IgG)	19 (41)
• Viral hepatitis serology	30 (65)
• Syphilis serology and/or rapid test	36 (78)
Imaging and minor procedures available *on site*, *n* (%)	
• Xray	23 (50)
• CT scan	20 (43)
• MRI	17 (37)
• Bronchoscopy	12 (26)
• Lumbar puncture	22 (48)
• Skin and soft tissue biopsy	23 (50)
• None	22 (48)
If blood tests or imaging or minor procedures not available *on site*, *n* = 32 (%)	
• Carried out in private sector	5 (16)
• Sent to the state laboratory	2 (6)
• A different reference hospital	25 (78)
Histopathology analysis available *on site*, *n* (%)	20 (43)
If not histopathology analysis available *on site*, *n* = 26 (%)	
• Carried out in private sector	1 (4)
• Sent to the state laboratory	2 (8)
• A different reference hospital	23 (88)
Clinical microbiology laboratory *on site*, *n* = 29 (%)	20 (43)
If not clinical microbiology laboratory *on site*,	
• Carried out in private sector	2 (7)
• Sent to the state laboratory	9 (31)
• A different reference hospital	18 (62)
Time to results when studies are sent somewhere else (blood tests, imaging, microbiology or histopathology analysis) days, median (IQR)	10 (7–25)
Microscope available *on site*, *n* (%)	28 (62)
Type of microscope, *n* = 28 (%)	
• Light	23 (82)
• Light and fluorescence	5 (18)
Direct microscopy assays available *on site*, *n* = 28 (%)	
• Direct cytology	23 (82)
• Gram staining	19 (68)
• KOH test	16 (57)
• Calcofluor white	5 (18)
• Silver staining/ Grocott	11 (39)
• Ziehl Neelsen staining	20 (71)
• None	5 (18)
Laminar flow hood available if clinical microbiology laboratory on site, *n* (%)	27 (59)
Clinical specimen that can be cultured at the local microbiology laboratory	
• Blood culture	32 (70)
• Bone marrow culture	18 (39)
• Urine cultures	33 (72)
• Spinal fluid cultures	28 (61)
• Bronchoalveolar lavages culture	23 (50)
• Biopsies culture	25 (54)
• None	13 (28)
Culture media available at the local microbiology laboratory	
• Blood agar	29 (63)
• Chocolate agar	24 (52)
• Sabouraud agar	21 (46)
• Potato dextrose agar	10 (22)
• CHROMagar ™	9 (20)
• MacConkey agar	21 (46)
• None	16 (35)
Local microbiology laboratory with mycobacteria culture capacity	16 (35)
Automation systems available at local laboratory, n (%)	18 (41)
Automation system available, *n* = 16 (%)	
• VITEK system (bioMerieux)	11 (69)
• BD Phoenix ™ (Becton Dickinson)	1 (6)
• MicroScan (Beckman Coulter)	1 (6)
• MALDI-TOF and VITEK	3 (19)
• MALDI-TOF	1 (6)
Molecular diagnostic testing locally available, n (%)	
• Homemade PCR testing	10 (22)
• GeneXpert (Cepheid)	23 (50)
• FilmArray ® (bioMerieux)	9 (20)
• None	21 (46)
Sequencing available *on site*, *n* (%)	4 (9)
• Sanger method	2 (50)

*CD4* Cluster of differentiation 4, *HIV* Human immunodeficiency virus, *CT scan* Computed tomography scan, *MRI* Magnetic resonance imaging, *IQR* Interquartile range, *KOH* Potassium hydroxide, *PCR* Polymerase chain reaction

Regarding access to diagnostic tests to identify fungal and other opportunistic infections, 25 centers reported having the capacity to diagnose cryptococcosis (54%) (Table 3, Fig. 2f). Excluding Indian ink test, only nine (20%) centers reported having either cryptococcal antigen (CrAg) or polymerase-chain reaction (PCR) to identify cryptococcosis (Table 3). Twenty-five centers in 16 states reported having local capacities to diagnose tuberculosis (54%) and 11 centers (24%) had access to aspergillosis or *Histoplasma* tests (Table 3, Fig. 2g). Seven centers (11%) distributed in 6 states reported having access to coccidioidomycosis tests. Only 2 of these 6 states are in the areas considered to be endemic for coccidioidomycosis (Fig. 2h). Finally, only five centers reported having access to *Pneumocystis* PCR (Fig. 2e, Table 3).

**Table 3:** Access to fungal diagnostic tests

Characteristic	*N* = 46 (%)
Mycology diagnostic testing available *on site*, n (%)	
Cryptococcosis	
• Cryptococcal antigen latex agglutination system	9 (20)
• Cryptococcal antigen (lateral flow assay)	2 (4)
• Cryptococcal PCR	3 (7)
• India ink	25 (54)
Aspergilosis	
• *Aspergillus* (Galactomannan) ELISA/EIA	11 (24)
• *Aspergillus* (Galactomannan) (lateral flow assay)	2 (4)
• *Aspergillus* serology (IgG/IgE)	6 (13)
Histoplasmosis	
• *Histoplasma* antigen (ELISA/EIA)	11 (24)
• *Histoplasma* antigen (lateral flow assay)	3 (7)
PCP and other	
• *Pneumocystis* PCR or IF Mab antibodies	5 (11)
• *Coccidioides* IgG/IgM, precipitins and/or ELISA	8 (17)
None	17 (37)
Antifungal susceptibility testing available *on site*, n (%)	15 (33)
• VITEK	13 (87)

*PCR* Polymerase chain reaction, *ELISA/EIA* Enzyme-linked immunosorbent assay/Enzyme immunoassay

The access to laboratory resources and diagnostic tests differed among health care level. Primary care centres attend to a larger number of PLWH compared to secondary and tertiary care institutions, including those with advanced disease. Contradictorily, primary care centers had less local diagnostic resources (Table 4). Regarding access to rapid tests in primary care centers only, the most accessible and widely available tests were syphilis serology and/or rapid test (66%), followed by viral hepatitis serology (45%), and *Mycobacterium tuberculosis* complex GeneXPert (41%). Toxoplasma serology was available in 21% of centers meanwhile CrAg by latex agglutination was available only in one primary care facility (3%), and Indian ink was available in 9 centers (31%). No primary care center had access to the lateral flow test for *Cryptococcus* or *Histoplasma* antigens. Regarding other diagnostic tests, only one primary health center was able to diagnose *Pneumocystis* pneumonia by PCR/IF, and two centers had access to serology for coccidioidomycosis, none of them are in the Mexican endemic region for coccidioidomycosis.

**Table 4:** Laboratory resources according to the healthcare level

Characteristic	Healthcare level			
	Total *n* = 46	Primary *n* = 29	Secondary *n* = 5	Tertiary *n* = 12
PLWH registered per centre, median (IQR)	773 (224 −1861)	1000 (404–2092)	244 (50–1000)	353 (210–1200)
PLWH attended per centre (monthly average 2020–2021), median (IQR)	200 (30–484)	350 (128 −738)	50 (12–120)	88 (23–180)
New registered PLWH per centre (monthly average 2020–2021), median (IQR),	10 (5–23)	15 (8–27)	7 (3–20)	6 (4–11)
New PLWH (<200 CD4 T-cells/mm^3^) per centre (monthly average 2020-2021), median (IQR)	7.5 (4–14)	6 (4–11)	8 (4–15)	7 (3–10)
Centre has a clinical laboratory on site (Yes), n (%)	25 (54)	8 (28)	5 (100)	12 (100)
Histopathology analysis available on site, n (%)	20 (43)	3 (10)	5 (100)	12 (100)
Clinical microbiology laboratory on site, *n* = 29 (%)	20 (43)	4 (14)	5 (100)	11 (92)
Blood tests and assays carried out on site, n (%)				
• Complete blood count	37 (80)	20 (69)	5 (100)	12 (100)
• Glucose and kidney function	36 (78)	19 (66)	5 (100)	12 (100)
• Liver function testing	32 (69)	16 (55)	5 (100)	11 (92)
• CD4 T cell counting	18 (39)	7 (24)	1 (20)	10 (83)
• HIV viral load	19 (41)	10 (34)	1 (20)	8 (67)
• Toxoplasma serology (IgM/IgG)	19 (41)	6 (21)	3 (60)	10 (83)
• Viral hepatitis serology	30 (65)	12 (45)	5 (100)	12 (100)
• Syphilis serology and/or rapid test	36 (78)	19 (66)	5 (100)	12 (100)
Microscope available on site, n (%)	28 (61)	11 (38)	5 (100)	12 (100)
Laminar flow hood	27 (59)	12 (41)	4 (80)	10 (92)
Sequencing available on site, n (%)	4 (9)	1 (3)	0 (0)	3 (25)
Method of susceptibility to antifungal agents	15 (33)	3 (10)	2 (40)	10 (83)
Mycobacteria diagnostic testing available on site, n (%)				
• GeneXpert	23 (50)	12 (41)	3 (60)	8 (67)
• Acid fast staining	23 (50)	4 (14)	4 (80)	12 (100)
• Mycobacterial cultures	16 (35)	8 (28)	2 (40)	6 (50)
Mycology diagnostic testing available on site, n (%)				
Cryptococcosis				
• Cryptococcal antigen latex agglutination system				8 (67)
• Cryptococcal antigen (lateral flow assay)	2 (4)	0 (0)	0 (0)	2 (17)
• Cryptococcal PCR	3 (7)	0 (0)	0 (0)	3 (25)
• India ink	25 (54)	9 (31)	4 (80)	12 (100)
Aspergilosis				
• *Aspergillus* (Galactomannan) ELISA/EIA	11 (24)	3 (10)	0 (0)	8 (67)
• *Aspergillus* (Galactomannan) (lateral flow assay)	2 (4)	0 (0)	0 (0)	2 (17)
• *Aspergillus *serology (IgG/IgE)	6 (13)	1 (4)	0 (0)	5 (42)
Histoplasmosis				
• *Histoplasma* antigen (ELISA/EIA)	11 (24)	4 (14)	1 (20)	6 (50)
• *Histoplasma* antigen (lateral flow assay)	3 (7)	0 (0)	0 (0)	2 (25)
PCP and other				
• *Pneumocystis* PCR or IF Mab antibodies	5 (11)	1 (3)	1 (20)	2 (25)
• *Coccidioides *IgG/IgM, precipitins and/or ELISA	7 (15)	2 (7)	0 (0)	5 (42)

*PLWH* People living with HIV, *IQR* Interquartile range, *CD4*  Cluster of differentiation 4, *PCR* Polymerase chain reaction, *IF* Immunofluorescent

## Discussion

The current study aimed to generate local data through a standardized questionnaire sent to representatives of primary, secondary, and tertiary health care institutions involved in HIV care. The obtained information helped to understand the level of access to diagnosis of IFD that PLWH have across the Mexican territory. We describe very low access to rapid tests and point-of-care tests in general in Mexican health centers, but more critically in primary care centers, where the utility of these tests is the highest.

Recently, articles have been published on the diagnostic capacities of fungal infections in Africa, Europe and Asia [9, 13, 14]. In Europe, a survey that included 45 countries reported overall good access to diagnostic tools and specifically to rapid tests, but with clear differences according to the country’s Growth Domestic Product [9]. A survey conducted by the European Confederation of Medical Mycology (ECMM) and the International Society for Human and Animal Mycology (ISHAM) explored access to diagnostic tools for IFDs in 40 countries and territories of the Asia Pacific Region [13]. Seventy-one percent of the included sites reported PLWH as their target patients. The study found that access to microscopy for IFD diagnosis was reported in 98.3% of the participating sites, and culture based methodologies in 97.4% of them. Additionally, between 60–70% of the centers had availability of CrAg and 21.7% for *Histoplasma* antigen detection [13]. A continent-wide survey showed that more than 70% of people in African countries had no public access to diagnostic tests for *Histoplasma* and *Pneumocystis* and 40% had no access to CrAg [15]. Cryptococcal antigen access was mostly provided by non-governamental organizations and other private health initiatives (15). The HIV-care funding situation in Latin America differs from other regions because Mexico and Latin American Countries in general obtain much less external non-governamental funding compared to some African regions [16]. In 2019, Latin America received a $3.03 billion USD from UNAIDS for HIV prevention and care, which is a less than a third of the amount for Southern Africa ($9.36 billion), and similar to $3.25 billion for the South Pacific Region [16].

Prior studies have evaluated local capacities to diagnose fungal infections in Latin America. One study reported a survey of 129 institutions in 14 countries in Latin America, 83% providing care for PLWH. Mexico participated with nine tertiary care centers. Urinary antigen for *Histoplasma* was available for 22% of the responders and 75% reported access to CrAg [17]. Another nation-wide survey from Honduras reported a very low access to fungal antigen detection tests, with only two institutions with access to *Histoplasma* antigen, and three institutions to CrAg lateral flow assay [18]. Many studies in Central America have described the positive impact of including rapid tests on the diagnosis yield of fungal diseases such as histoplasmosis and cryptococcosis [19, 20].

In Guatemala, implementing rapid antigen tests for histoplasmosis improved more than double the diagnostic capacity compared to the exclusive use cultures [21]. Besides an improvement in case detection, there is also evidence that prompt diagnosis of opportunistic infections such as cryptococcosis and histoplasmosis through a rapid test improves survival [20, 22]. *Paccoud *et al. evaluated the impact in mortality of prior screening with CrAg, in PLWH with a CD4 count < 100 cells/mm3 and confirmed meningeal cryptococosis or cryptococcemia. They found a 17% reduction of mortality in patients screened compared to patients not screened [20]. Medina et al., described the impact of implementing a program of prospective screening for OI in patients with advanced disease in Guatemala. After one year of the implementation, they found a 7% reduction in mortality and 5% increase in targeted treatment for OIs [22]. Rajasingham et al., in their decision analytical model, evaluated histoplasma antigen screening in PLWH with advanced disease, and report that routine screening avoids an estimated 17% deaths in cases of advanced HIV disease [23].

Among PLWH, fungal diseases remain a major cause of morbidity and mortality, mainly due to meningeal cryptococcosis and disseminated histoplasmosis. Thanks to the implementation of screening programs using rapid tests, two recent studies carried out in Guatemala demonstrated that these two IFD have a higher incidence than previously estimated [22, 24, 25]. Coccidioidomycosis is considered endemic in Northern Mexico, histoplasmosis in Mid-Southern Mexico, and cryptococcosis represents one of the three most serious fungal infections in PLWH [26, 27]. These data support the need to have rapid access to identify these fungal etiologies.

We also provide data regarding access to diagnosis of other potentially deadly OI such as mycobacterial infections, including tuberculosis (TB). Tuberculosis was the OI with the highest diagnostic capacity, mainly due to access to GeneXpert (Cepheid), however the diagnostic capacity remained low, at 50%. In a prospective cohort study including patients coinfected with HIV/TB from four different regions of the world (Eastern Europe (EE), Western Europe (WE), Southern Europe (SE) and Latin America (LA)), the proportion of patients with definite TB diagnosis in EE (47%) and LA (40%) was lower than WE (71%) and SE (72%), reflecting poor access to diagnostic tests in those regions [28]. These were also the regions with the lowest median CD4 + T cell count. Although the national TB incidence rate is estimated at 25 cases per 100,000 population [29], this varies widely by geographical region, with highest burden in the poorest regions, and usually in regions with higher HIV/TB coinfection.

We report eight states with more than 50% advanced disease among individuals with new HIV diagnosis. These proportions are similar to the official report on the proportion of patients with < 200 CD4 + T cells by the National Center for Prevention and Control of HIV/AIDS (CENSIDA) [4]. Most of these states are located either at the border with the USA (two states), or the southern region of the country (four states). This latter region is characterized by poorer socioeconomic conditions, with higher proportion of informal labor and indigenous population, and lower education [30]. It is also the region with the highest burden of fungal diseases and should be the region with better access to rapid detection tools. Instead, only one center of the southern area was able to diagnose histoplasmosis and cryptococcosis with rapid tests.

Regarding hepatitis C, we report access to hepatitis serology in less than 65% of the centers: 100% of second and tertiary centers, 45% in primary care facilities. This is of central importance as recently, a nationwide hepatitis C program was implemented by the Mexican government aiming to provide free access to direct-acting antivirals [31]. Certainly, the success in implementing this and any other program will rely on having adequate screening tools at all health care levels and not only at tertiary care institutions. However, it is possible that our survey does not reflect all centers currently screening for hepatitis C, since the number of centers in the program has increased in the last two years.

It is important mentioning that half of the centers has at least one need to send their samples for analysis to another facility. Relying on external laboratories results in long waiting times, reflecting how the lack of local diagnostic resources impacts and delays diagnostic and treatment.

Our study has some limitations. Due to the nature of the study, we reported only information from the centers who replied to our request, and our response rate was 57%. These data might not be representative of every state in the country, particularly for the states where we did not get a reply. For the states who replied with only one or two answers, we generalized the information of the state from only a few answers, and there could also be differences intra-state that could not be perceived in this report. However, we managed to describe the situation for almost three quarters of the states, and we had representation of the poorest states of the country with the highest burden of OIs, as well as representation from the centers with greater capacity of HIV care.

In conclusion, in Mexico, most public HIV-dedicated health care centers lack on-site capacity to diagnose opportunistic infections, specifically fungal infections. Rapid tests and point-of-care tests are frequently unavailable, which is more pronounced in primary care centres. Considering that IFDs still contribute significantly to mortality among PLWH, better access to diagnostic tools in all levels of HIV-care is urgent.

## Supplementary Information

Supplementary Material 1.

Supplementary Material 2.

Supplementary Material 3.

## Data Availability

All data is available in this document and supplementary material. Any further data can be provided upon request to the authors.

## Abbreviations

OI: Opportunistic infections
PLWH: People living with HIV
IFD: Invasive fungal diseases
LMIC: Low- and middle-income countries
WHO: World Health Organization
CrAg: Cryptococcal Antigen
PCR: Polymerase chain reaction
EE: Eastern Europe
WE: Western Europe
SE: Southern Europe
LA: Latin America
