# Supplementary material for: Access to diagnostic testing for invasive fungal diseases and other opportunistic infections in Mexican health care centers caring for patients living with HIV
Source: BMC Health Serv Res. 2025 Feb 19;25:275. doi: 10.1186/s12913-025-12405-5 (PMC11837643; doi:10.1186/s12913-025-12405-5)
Supplement: Supplementary file 1 — Supplementary Material 1. [file 12913_2025_12405_MOESM1_ESM.docx]

**Access to Diagnosis of Fungal and Other Opportunistic Diseases for Individuals Living with HIV**

This survey was designed through a collaboration of the Coordinating Commission of National Health Institutes and High-Specialty Hospitals (CCINSHAE), the Mexican Association of Infectious Diseases and Microbiology (AMIMC), HIV Chapter, and the National Center for HIV/AIDS Prevention and Control (CENSIDA)

We will now ask you questions about the capacity of your center(s) to diagnose opportunistic infections in people living with HIV.

**Section 1: General Information**

Write your response below each question.

1. Full Name of the Respondent _____________
2. Contact Email ________________
3. Name of the Center __________________
4. Center Location (Municipality, State) ___________________
5. Number of centers for which you will report responses __________________

**Section 2: General Characteristics**

1. Total Population (number) of People Living with HIV (PLHIV) in care at your center (active patients) _______________
2. Number of PLHIV receiving care monthly in your center(s) (average monthly 2020-2021) ________________
3. Number of new PLHIV in your center(s) per month (average monthly 2020-2021) __________
4. Number of new PLHIV in your center per month with <200 CD4 count (average monthly 2020-2021) _______________
5. Does your center have the capacity to diagnose new patients with HIV and opportunistic diseases? __________________

- Yes, both
- Only new HIV patients
- None

1. Does your center have a clinical laboratory?
   Select only one option.

- Yes
- No

1. If the answer to question 11 was "No," indicate where you send your samples for diagnosis. _____________
2. If your answer to question 11 was "Yes," indicate all test available at your local laboratory:

- Complete Blood Count
- Blood Chemistry
- Liver Function Tests
- CD4 Count
- HIV Viral Load
- Toxoplasma IgM/IgG
- Viral Hepatitis Profile
- Syphilis Serology/Rapid Test

1. Does your care center have the following services or perform the following procedures? (Check all those available)

- X-rays
- CT Scan
- MRI
- Bronchoscopy
- Lumbar Puncture
- Biopsies
- None

1. If any or all responses in question 14 are negative, indicate where the services/procedures are performed if needed. _____________
2. Does your care center have a histopathology/pathology service?
   Select only one option.

- Yes
- No

1. If the answer to question 16 is "No," indicate where services/procedures are performed if needed._____________
2. Does your center have a microbiology laboratory?
   Select only one option.

- Yes
- No

1. If the answer to question 18 was "No," indicate where you send your tests. You may list more than one location. ____________
2. If the answer to question 18 was "No," indicate approximate result turnaround time (average, minimum, and maximum in days). ___________________

**Section 3: Diagnostic Capacities**

1. Does your care center have a microscope?
   Select only one option.

- Yes
- No

1. Type of microscope available
   Select only one option.

- Conventional
- Fluorescence
- Both

1. If you have a microscope, what microscopic exams are performed?
   Select all that apply.

- Direct Cytology
- Gram Stain
- KOH
- Calcofluor
- Silver or Grocott Stain
- Ziehl Neelsen Stain
- None

1. If your center has access to a local microbiology and/or clinical analysis laboratory, is there a laminar flow hood?
   Select only one option.

- Yes
- No

1. If your center has access to a local microbiology and/or clinical analysis laboratory, indicate which of the following are available:
   Check all of those available

- Blood Cultures
- Bone Marrow Cultures
- Urine Cultures
- CSF Cultures
- Bronchoalveolar Lavage Cultures
- Biopsy Cultures
- None

1. If your center has access to a local microbiology and/or clinical analysis laboratory, are culture media available?
   Check all those available.

- Blood Agar
- Chocolate Agar
- Sabouraud
- Potato Dextrose Agar
- Chrom Agar
- MacConkey Agar
- None

1. If your center has access to a local microbiology and/or clinical analysis laboratory, is there the possibility to culture Mycobacteria?
   Select only one option.

- Yes
- No

1. If your center has access to a local microbiology and/or clinical analysis laboratory, is there an automated identification method (e.g., Vitek, MALDI-ToF)?
   Select only one option.

- Yes
- No

1. If your answer in question 28 was "Yes," please specify which method.

_______________.

1. If your center has access to a local microbiology and/or clinical analysis laboratory, is there a molecular identification method?
   Select all of those available

- In-house PCR
- Genexpert
- Filmarray
- Other commercial platform
- None

1. If your center has access to a local microbiology and/or clinical analysis laboratory, is there a sequencing method (e.g., Sanger)?
   Select only one option.

- Yes
- No

1. If your answer in question 31 was "Yes," please specify which method.

____________________

**Section 4: Diagnostic Capacities in Mycology**

1. If your center has access to a local microbiology and/or clinical analysis laboratory, are the following mycological diagnostic tests available?
   Select all of those available

- Cryptococcal Antigen (latex agglutination)
- Rapid Test for Cryptococcus (lateral flow device)
- PCR for Cryptococcus
- India Ink Test
- Aspergillus Antigen (Galactomannan) ELISA/EIA
- Aspergillus Antigen (Galactomannan) (lateral flow device)
- Histoplasma Antigen (ELISA/EIA)
- Histoplasma Antigen (lateral flow device)
- PCR or serology for Pneumocystis
- IgG/IgE for Aspergillus
- IgG/IgM, precipitin, and/or ELISA for Coccidioidomycosis
- None

1. If your center has access to a local microbiology and/or clinical analysis laboratory, is there an antifungal susceptibility method?
   Select only one option.

- Yes
- No

1. If your answer in question 34 was "Yes," please specify which method (e.g., VITEK).

**THANK YOU FOR ANSWERING THIS SURVEY**
