# Supplementary material for: Access to diagnostic testing for invasive fungal diseases and other opportunistic infections in Mexican health care centers caring for patients living with HIV
Source: BMC Health Serv Res. 2025 Feb 19;25:275. doi: 10.1186/s12913-025-12405-5 (PMC11837643; doi:10.1186/s12913-025-12405-5)
Supplement: Supplementary file 2 — Supplementary Material 2. [file 12913_2025_12405_MOESM2_ESM.docx]

**Supplement Table S1 : diagnostic test capacity for each infection, by state***

| **State/Disease** | **Syphilis** | **Toxoplasmosis** | **Hepatitis** | **Tuberculosis** | | **PJP** | | **Cryptococcosis** | | | **Histoplasmosis** | | **Coccidioidomicosis** |
| --- | --- | --- | --- | --- | --- | --- | --- | --- | --- | --- | --- | --- | --- |
|  | **Serology/**  **rapid test** | **Serology igG/igM** |  | **GeneXpert** | **Other (culture/ZN)** | **PCR and/or IF antibodies and silver staining** | **Silver staining** | **India ink only** | **India ink and latex agglutination** | **India ink, latex agglutination and either PCR or LFA** | **ELISA/EIA histoplasma** | **LFA histoplasma** | **IgM/IgG precipitins** |
| Aguascalientes  n =1 | 1 (100) | 0 | 1 (100) | 1 (100) | 0 | 1 (100) | 0 | 0 | 0 | 0 | 1 (100) | 0 | 1 (100) |
| Baja California  n =2 | 2 (100 | 0 | 1 (50) | 1 (50) | 0 | 0 | 0 | 0 | 0 | 0 | 0 | 0 | 0 |
| Campeche  n=1 | 1 (100) | 0 | 0 | 0 | 0 | 0 | 0 | 0 | 0 | 0 | 0 | 0 | 0 |
| Mexico City  n=6 | 6 (100) | 2 (33) | 6 (100) | 6 (100) | 0 | 1 (17) | 3 (50) | 3 (50) | 1 (17) | 1 (17) | 3 (50) | 2 (33) | 2 (33) |
| **Chiapas**  **n=3** | **3 (100)** | **3 (100)** | **3 (100)** | **2 (67)** | **0** | **0** | **1 (33)** | **2 (67)** | **1 (33)** | **0** | **1 (33)** | **0** | **1 (33)** |
| Chihuahua  n=1 | 1 (100) | 0 | 0 | 0 | 0 | 0 | 0 | 1 (100) | 0 | 0 | 0 | 0 | 0 |
| **Durango**  **n=1** | **1 (100)** | **0** | **1 (100)** | **0** | **0** | **0** | **0** | **1 (100)** | **0** | **0** | **0** | **0** | **0** |
| Estado de México  n=2 | 1 (50) | 1 (50) | 1 (350) | 0 | 1 (50) | 0 | 1 (50) | 0 | 1 (50) | 0 | 1 (50) | 0 | 1 (50) |
| Guanajuato  n=9 | 6 (67) | 3 (33) | 5 (56) | 4 (44) | 1 (9) | 1 (11) | 1 (11) | 2 (22) | 0 | 1 (11) | 3 (33) | 1 (33) | 1 (33) |
| Guerrero  n=1 | 1 (100) | 0 | 0 | 1 (100) | 0 | 0 | 0 | 1 (100) | 0 | 0 | 0 | 0 | 0 |
| **Hidalgo**  **n=1** | **1 (100)** | **1 (100)** | **1 (100)** | **0** | **0** | **0** | **0** | **0** | **0** | **0** | **0** | **0** | **0** |
| Jalisco  n=3 | 2 (67) | 2 (67) | 3 (100) | 3 (100) | 0 | 1 (33) | 2 (67) | 2 (67) | 0 | 0 | 1 (33) | 0 | 0 |
| **Michoacán**  n=1 | **1 (100)** | **1 (100)** | **1 (100)** | **0** | **1 (100)** | **0** | **1 (100)** | **1 (100)** | **0** | **0** | **0** | **0** | **0** |
| Morelos  n=1 | 0 | 0 | 0 | 1 (100) | 0 | 0 | 0 | 0 | 0 | 0 | 0 | 0 | 0 |
| **Nuevo León**  **n=1** | **1 (100)** | **1 (100)** | **1 (100)** | **1 (100** | **0** | **1 (100)** | **1 (100)** | **0** | **0** | **1 (100)** | **0** | **0** | **1 (100)** |
| **Oaxaca**  n=2 | **2 (100)** | **1 (50)** | **2 (100)** | **1 (50)** | **1 (50)** | **0** | **1 (50)** | **0** | **1 (50)** | **0** | **0** | **0** | 0 |
| Puebla  n=1 | 0 | 0 | 0 | 0 | 0 | 0 | 0 | 0 | 0 | 0 | 0 | 0 | 0 |
| Quintana Roo  n=1 | 1 (100) | 0 | 0 | 0 | 0 | 0 | 0 | 0 | 0 | 0 | 0 | 0 | 0 |
| San Luis Potosí  n=1 | 1 (100) | 1 (100) | 1 (100) | 1 (100) | 0 | 0 | 0 | 0 | 1 (100) | 0 | 0 | 0 | 0 |
| Sonora  n=3 | 1 (33) | 0 | 0 | 1 (33) | 1 (33) | 0 | 0 | 0 | 0 | 0 | 1 (33) | 0 | 1 (33) |
| Tabasco  n=1 | 0 | 0 | 0 | 0 | 0 | 0 | 0 | 0 | 0 | 0 | 0 | 0 | 0 |
| **Veracruz**  n=2 | **2 (100)** | **2 (100)** | **2 (100** | **0** | **1 (50)** | **0** | **0** | **2 (100)** | **0** | **0** | **0** | **0** | **0** |
| **Yucatán**  n=1 | **1 (100)** | **1 (100)** | **1 (100)** | **0** | **1 (100)** | **0** | **0** | **1 (100)** | **0** | **0** | **0** | **0** | **0** |

***results are reported by answer, not by center (n=46)**

**Esta me parece que no se debe incluir**

| **State** | **>40% dx tardío (base de datos)** | **n (%)** | **>40% dx tardío (boletín censida)** | **%** |
| --- | --- | --- | --- | --- |
| Aguascalientes  n=60 | SI | 30 (50) | NO | 18 |
| Baja California  n=35 | SI | 15 (42.8) | NO | 38 |
| Campeche  n= 10 | SI | 5 (50) | SI | 44 |
| Chiapas  n=28 | SI | 24 (85.7) | SI | 48 |
| Chihuahua  n=25 | SI | 12 (48) | NO | 39 |
| Mexico City  n=427 | NO | 167 (39.6) | NO | 26 |
| Durango  n=3 | SI | 2 (66.7) | NO | 37 |
| Estado de México  n=23 | SI | 11 (47.8) | SI | 42 |
| Guanajuato  n= 53 | SI | 24 (45.2) | NO | 26 |
| Guerrero  n=15 | SI | 7 (46.7) | NO | 21 |
| Hidalgo  n=23 | SI | 14 (60.9) | SI | 41 |
| Jalisco  n=70 | SI | 45 (64.3) | NO | 34 |
| Michoacán  n=3 | SI | 3 (100) | NO | 34 |
| Morelos  n =25 | SI | 10 (40) | NO | 38 |
| Nuevo León  N =10 | SI | 9 (90) | SI | 49 |
| Oaxaca  n=54 | NO | 38 (70.3) | SI | 58 |
| Puebla  n=100 | SI | 40 (40) | NO | 29 |
| Quintana Roo  n=60 | SI | 42 (40) | NO | 35 |
| San Luis Potosí  n=16 | SI | 8 (50) | NO | 35 |
| Sonora  n= 15 | NO | 4 (26.6) | NO | 24 |
| Tabasco  n= 15 | SI | 8 (53.3) | NO | 28 |
| Veracruz  n=60 | SI | 40 (75) | SI | 52 |
| Yucatán  n=5 | SI | 5 (100) | NO | 25 |

| **Característica** | **Baja California**  **n=2** | **CDMX**  **n=6** | **Chiapas**  **n=3** | **Edo Mex**  **n=2** | **Guanajuato**  **n=9** | **Jalisco**  **n =3** | **Oaxaca**  **n=2** | **Veracruz**  **n=2** |
| --- | --- | --- | --- | --- | --- | --- | --- | --- |
| Laboratorio de análisis clínicos | 1 (50) | 6 (100) | 3 (100) | 1 (50) | 3 (33) | 2 (67) | 2 (100) | 2 (100) |
| Histopatología/anatomía patológica | 1 (50) | 4 (67) | 3 (100) | 1 (50) | 2 (22) | 2 (67) | 1 (50) | 2 (100) |
| Laboratorio de microbiología | 1 (50) | 4 (67) | 3 (100) | 0 | 3 (33) | 2 (67) | 1 (50) | 2 (100) |
| Cuenta con microscopio | 1 (50) | 6 (100) | 3 (100) | 1 (50) | 3 (33) | 3 (100) | 2 (100) | 2 (100) |
| Campana de flujo laminar | 2 (100) | 6 (100) | 1 (33) | 0 | 3 (33) | 3 (100) | 2 (100) | 2 (100) |
| Método de Secuenciación | 0 | 3 (50) | 0 | 0 | 0 | 0 | 0 | 0 |
| Método de susceptibilidad a antifúngicos | 1 (50) | 4 (67) | 3 (100) | 0 | 1 (11) | 1 (33) | 1 (50) | 1 (50) |
| Capacidad para detectar micobacterias  Rápida   - GeneXpert - ZN   Tradicional   - Cultivo de micobacterias | 1 (50)  1 (50)  1 (50) | 6 (100)  5 (83)  3 (50) | 2 (67)  2 (67)  0 | 0  1(50)  1 (50) | 4 (44)  2 (22)  4 (44) | 3 (100)  3 (100)  2 (67) | 1 (50)  1 (50)  0 | 0  1 (50)  0 |
|  |  |  |  |  |  |  |  |  |
| Capacidad para detectar Criptococo  Rápida   - Antígeno de Criptococo (aglutinación en látex) - Prueba rápida para Criptococo (dispositivo de flujo lateral) - Tinta china   Tradicional   - PCR para Criptococco | 0  0  0  0 | 3 (50)  1 (17)  6 (100)  1 (17) | 1 (33)  0  3 (100)  0 | 1 (50)  0  1 (50)  0 | 1 (11)  1 (11)  3 (33)  1 (11) | 0  0  2 (67)  0 | 1 (50)  0  1 (50)  0 | 0  0  2 (100)  0 |
|  |  |  |  |  |  |  |  |  |
|  |  |  |  |  |  |  |  |  |
| Capacidad para detectar Aspergillus  Rápida   - Antígeno de Aspergillus (Galactomano) ELISA/EIA - Antígeno de Aspergillus (Galactomano) (dispositivo de flujo lateral)   Tradicional   - IgG/IgE para Aspergillus | 0  0  0 | 3 (50)  1 (17)  2 (33) | 1 (33)  0  1 (33) | 1 (50)  0  1 (50) | 1 (11)  1 (11)  1 (11) | 0  0  0 | 0  0  0 | 0  0  0 |
|  |  |  |  |  |  |  |  |  |
|  |  |  |  |  |  |  |  |  |
| Capacidad para detectar Histoplasma  Rápida   - Antígeno de Histoplasma (ELISA/EIA) - Antígeno de Histoplasma (dispositivo de flujo lateral) | 0  0 | 3 (50)  2 (33) | 1 (33)  0 | 1 (50)  0 | 3 (33)  1 (11) | 1 (33)  0 | 0  0 | 0  0 |
|  |  |  |  |  |  |  |  |  |
| PCR o anticuerpos contra Pneumocystis | 0 |  | 0 | 0 | 1 (11) | 1 (33) | 0 | 0 |
| IgG/IgM, precipitinas y/o ELISA para Coccidioidomicosis | 0 |  | 1 (33) | 1 (50) | 1 (11) | 0 | 0 | 0 |
