# Supplementary material for: Access to diagnostic testing for invasive fungal diseases and other opportunistic infections in Mexican health care centers caring for patients living with HIV
Source: BMC Health Serv Res. 2025 Feb 19;25:275. doi: 10.1186/s12913-025-12405-5 (PMC11837643; doi:10.1186/s12913-025-12405-5)
Supplement: Supplementary file 3 — Supplementary Material 3. [file 12913_2025_12405_MOESM3_ESM.docx]

**Supplement Table S1 : diagnostic test capacity for each infection, by state***

| **State/Disease** | **Syphilis** | **Toxoplasmosis** | **Hepatitis** | **Tuberculosis** | | **PJP** | | **Cryptococcosis** | | | **Histoplasmosis** | | **Coccidioidomicosis** |
| --- | --- | --- | --- | --- | --- | --- | --- | --- | --- | --- | --- | --- | --- |
|  | **Serology/**  **rapid test** | **Serology igG/igM** |  | **GeneXpert** | **Other (culture/ZN)** | **PCR and/or IF antibodies and silver staining** | **Silver staining** | **India ink only** | **India ink and latex agglutination** | **India ink, latex agglutination and either PCR or LFA** | **ELISA/EIA histoplasma** | **LFA histoplasma** | **IgM/IgG precipitins** |
| Aguascalientes  n =1 | 1 (100) | 0 | 1 (100) | 1 (100) | 0 | 1 (100) | 0 | 0 | 0 | 0 | 1 (100) | 0 | 1 (100) |
| Baja California  n =2 | 2 (100 | 0 | 1 (50) | 1 (50) | 0 | 0 | 0 | 0 | 0 | 0 | 0 | 0 | 0 |
| Campeche  n=1 | 1 (100) | 0 | 0 | 0 | 0 | 0 | 0 | 0 | 0 | 0 | 0 | 0 | 0 |
| Mexico City  n=6 | 6 (100) | 2 (33) | 6 (100) | 6 (100) | 0 | 1 (17) | 3 (50) | 3 (50) | 1 (17) | 1 (17) | 3 (50) | 2 (33) | 2 (33) |
| **Chiapas**  **n=3** | **3 (100)** | **3 (100)** | **3 (100)** | **2 (67)** | **0** | **0** | **1 (33)** | **2 (67)** | **1 (33)** | **0** | **1 (33)** | **0** | **1 (33)** |
| Chihuahua  n=1 | 1 (100) | 0 | 0 | 0 | 0 | 0 | 0 | 1 (100) | 0 | 0 | 0 | 0 | 0 |
| **Durango**  **n=1** | **1 (100)** | **0** | **1 (100)** | **0** | **0** | **0** | **0** | **1 (100)** | **0** | **0** | **0** | **0** | **0** |
| Estado de México  n=2 | 1 (50) | 1 (50) | 1 (350) | 0 | 1 (50) | 0 | 1 (50) | 0 | 1 (50) | 0 | 1 (50) | 0 | 1 (50) |
| Guanajuato  n=9 | 6 (67) | 3 (33) | 5 (56) | 4 (44) | 1 (9) | 1 (11) | 1 (11) | 2 (22) | 0 | 1 (11) | 3 (33) | 1 (33) | 1 (33) |
| Guerrero  n=1 | 1 (100) | 0 | 0 | 1 (100) | 0 | 0 | 0 | 1 (100) | 0 | 0 | 0 | 0 | 0 |
| **Hidalgo**  **n=1** | **1 (100)** | **1 (100)** | **1 (100)** | **0** | **0** | **0** | **0** | **0** | **0** | **0** | **0** | **0** | **0** |
| Jalisco  n=3 | 2 (67) | 2 (67) | 3 (100) | 3 (100) | 0 | 1 (33) | 2 (67) | 2 (67) | 0 | 0 | 1 (33) | 0 | 0 |
| **Michoacán**  n=1 | **1 (100)** | **1 (100)** | **1 (100)** | **0** | **1 (100)** | **0** | **1 (100)** | **1 (100)** | **0** | **0** | **0** | **0** | **0** |
| Morelos  n=1 | 0 | 0 | 0 | 1 (100) | 0 | 0 | 0 | 0 | 0 | 0 | 0 | 0 | 0 |
| **Nuevo León**  **n=1** | **1 (100)** | **1 (100)** | **1 (100)** | **1 (100** | **0** | **1 (100)** | **1 (100)** | **0** | **0** | **1 (100)** | **0** | **0** | **1 (100)** |
| **Oaxaca**  n=2 | **2 (100)** | **1 (50)** | **2 (100)** | **1 (50)** | **1 (50)** | **0** | **1 (50)** | **0** | **1 (50)** | **0** | **0** | **0** | 0 |
| Puebla  n=1 | 0 | 0 | 0 | 0 | 0 | 0 | 0 | 0 | 0 | 0 | 0 | 0 | 0 |
| Quintana Roo  n=1 | 1 (100) | 0 | 0 | 0 | 0 | 0 | 0 | 0 | 0 | 0 | 0 | 0 | 0 |
| San Luis Potosí  n=1 | 1 (100) | 1 (100) | 1 (100) | 1 (100) | 0 | 0 | 0 | 0 | 1 (100) | 0 | 0 | 0 | 0 |
| Sonora  n=3 | 1 (33) | 0 | 0 | 1 (33) | 1 (33) | 0 | 0 | 0 | 0 | 0 | 1 (33) | 0 | 1 (33) |
| Tabasco  n=1 | 0 | 0 | 0 | 0 | 0 | 0 | 0 | 0 | 0 | 0 | 0 | 0 | 0 |
| **Veracruz**  n=2 | **2 (100)** | **2 (100)** | **2 (100** | **0** | **1 (50)** | **0** | **0** | **2 (100)** | **0** | **0** | **0** | **0** | **0** |
| **Yucatán**  n=1 | **1 (100)** | **1 (100)** | **1 (100)** | **0** | **1 (100)** | **0** | **0** | **1 (100)** | **0** | **0** | **0** | **0** | **0** |

***results are reported by answer, not by center (n=46)**

Supplement table S2: General Diagnostic test capacity by state*

| Characteristic | Baja California  n=2 | CDMX  n=6 | Chiapas  n=3 | Edo Mex  n=2 | Guanajuato  n=9 | Jalisco  n =3 | Oaxaca  n=2 | Veracruz  n=2 |
| --- | --- | --- | --- | --- | --- | --- | --- | --- |
| Clinical laboratory | 1 (50) | 6 (100) | 3 (100) | 1 (50) | 3 (33) | 2 (67) | 2 (100) | 2 (100) |
| Histopathology/ pathology service | 1 (50) | 4 (67) | 3 (100) | 1 (50) | 2 (22) | 2 (67) | 1 (50) | 2 (100) |
| Microbiology laboratory | 1 (50) | 4 (67) | 3 (100) | 0 | 3 (33) | 2 (67) | 1 (50) | 2 (100) |
| Microscope available | 1 (50) | 6 (100) | 3 (100) | 1 (50) | 3 (33) | 3 (100) | 2 (100) | 2 (100) |
| Laminar Flow hood available | 2 (100) | 6 (100) | 1 (33) | 0 | 3 (33) | 3 (100) | 2 (100) | 2 (100) |
| Sequencing method available | 0 | 3 (50) | 0 | 0 | 0 | 0 | 0 | 0 |
| Antifungal susceptibility | 1 (50) | 4 (67) | 3 (100) | 0 | 1 (11) | 1 (33) | 1 (50) | 1 (50) |
| Rapid tests for mycobacterial detection   - GeneXpert - ZN   Traditional detection tests   - Mycobacterial culture | 1 (50)  1 (50)  1 (50) | 6 (100)  5 (83)  3 (50) | 2 (67)  2 (67)  0 | 0  1(50)  1 (50) | 4 (44)  2 (22)  4 (44) | 3 (100)  3 (100)  2 (67) | 1 (50)  1 (50)  0 | 0  1 (50)  0 |
|  |  |  |  |  |  |  |  |  |
| Rapid tests for cryptococcal detection   - Cryptococccal Antigen (latex agglutination) - Cryptococcal antigen (lateral flow device) - Indian ink   Other tests for Cryptococci   - PCR for Cryptococci | 0  0  0  0 | 3 (50)  1 (17)  6 (100)  1 (17) | 1 (33)  0  3 (100)  0 | 1 (50)  0  1 (50)  0 | 1 (11)  1 (11)  3 (33)  1 (11) | 0  0  2 (67)  0 | 1 (50)  0  1 (50)  0 | 0  0  2 (100)  0 |
|  |  |  |  |  |  |  |  |  |
|  |  |  |  |  |  |  |  |  |
| Rapid tests for Aspergillus detection   - Aspergillus Antigen (Galactomannan) ELISA/EIA - Aspergillus Antigen (Galactomannan) (Lateral flor device)   Other tests for Aspergillus   - IgG/IgE for Aspergillus | 0  0  0 | 3 (50)  1 (17)  2 (33) | 1 (33)  0  1 (33) | 1 (50)  0  1 (50) | 1 (11)  1 (11)  1 (11) | 0  0  0 | 0  0  0 | 0  0  0 |
|  |  |  |  |  |  |  |  |  |
|  |  |  |  |  |  |  |  |  |
| Rapid tests for Histoplasma   - Histoplasma Antigen (ELISA/EIA) - Histoplasma Antigen (lateral flow device) | 0  0 | 3 (50)  2 (33) | 1 (33)  0 | 1 (50)  0 | 3 (33)  1 (11) | 1 (33)  0 | 0  0 | 0  0 |
|  |  |  |  |  |  |  |  |  |
| PCR or Serology for Pneumocystis | 0 |  | 0 | 0 | 1 (11) | 1 (33) | 0 | 0 |
| IgG/IgM, precipitins and/or ELISA for Coccidioidomycosis | 0 |  | 1 (33) | 1 (50) | 1 (11) | 0 | 0 | 0 |

*only states with more than one answer are included
